# MicroRNA-449a Overexpression, Reduced NOTCH1 Signals and Scarce Goblet Cells Characterize the Small Intestine of Celiac Patients

**DOI:** 10.1371/journal.pone.0029094

**Published:** 2011-12-15

**Authors:** Marina Capuano, Laura Iaffaldano, Nadia Tinto, Donatella Montanaro, Valentina Capobianco, Valentina Izzo, Francesca Tucci, Giancarlo Troncone, Luigi Greco, Lucia Sacchetti

**Affiliations:** 1 CEINGE (Centro di Ingegneria Genetica) Advanced Biotechnology, s. c. a r. l., Naples, Italy; 2 Department of Biochemistry and Medical Biotechnology, University of Naples Federico II, Naples, Italy; 3 Fondazione IRCSS SDN (Istituto di Ricovero e Cura a Carattere Scientifico - Istituto di Ricerca Diagnostica e Nucleare), Naples, Italy; 4 Department of Paediatrics and European Laboratory for the Investigation of Food-Induced Diseases (ELFID), University of Naples Federico II, Naples, Italy; 5 Department of Biomorphological and Functional Sciences, University of Naples Federico II, Naples, Italy; Emory University, United States of America

## Abstract

MiRNAs play a relevant role in regulating gene expression in a variety of physiological and pathological conditions including autoimmune disorders. MiRNAs are also important in the differentiation and function of the mouse intestinal epithelium. Our study was aimed to look for miRNA-based modulation of gene expression in celiac small intestine, and particularly for genes involved in cell intestinal differentiation/proliferation mechanisms. A cohort of 40 children (20 with active CD, 9 on a gluten-free diet (GFD), and 11 controls), were recruited at the Paediatrics Department (University of Naples Federico II). The expression of 365 human miRNAs was quantified by TaqMan low-density arrays. We used bioinformatics to predict putative target genes of miRNAs and to select biological pathways. The presence of NOTCH1, HES1, KLF4, MUC-2, Ki67 and beta-catenin proteins in the small intestine of CD and control children was tested by immunohistochemistry. The expression of about 20% of the miRNAs tested differed between CD and control children. We found that high miR-449a levels targeted and reduced both NOTCH1 and KLF4 in HEK-293 cells. NOTCH1, KLF4 signals and the number of goblet cells were lower in small intestine of children with active CD and in those on a GFD than in controls, whereas more nuclear beta-catenin staining, as a sign of the WNT pathway activation, and more Ki67 staining, as sign of proliferation, were present in crypts from CD patients than in controls.

In conclusion we first demonstrate a miRNA mediated gene regulation in small intestine of CD patients. We also highlighted a reduced NOTCH1 pathway in our patients, irrespective of whether the disease was active or not. We suggest that NOTCH pathway could be constitutively altered in the celiac small intestine and could drive the increased proliferation and the decreased differentiation of intestinal cells towards the secretory goblet cell lineage.

## Introduction

Celiac disease (CD) is an immunomediated enteropathy and one of the most heritable complex diseases. The concordance rate in monozygotic twins is 75% [1], [2]. HLA DQ2/DQ8 haplotypes confer the highest estimated heritability (∼35%) [3] reported so far.

Exposure to gliadin triggers an inappropriate immune response in HLA-susceptible individuals. However, the presence of HLA-risk alleles is a necessary but not sufficient condition for the development of CD. In fact, about 30–40% of healthy subjects carry HLA-risk alleles [4], [5]. Attempts at identifying non-HLA major genetic risk loci have been unsuccessful [6]. Gluten has also been shown to affect epithelial differentiation-associated genes in the small intestinal mucosa of celiac patients [7], [8]. However, the role of miRNA-based regulatory mechanisms in mediating gene expression alteration in CD has not yet been investigated.

MicroRNAs (miRNAs) are small non-coding RNAs, 20–25 nt long, that modulate gene expression through canonical base pairing to complementary sequences in the 3′UTR of target mRNA [9]. Since their identification in 1993 [10], miRNAs have been found to play a relevant role in regulating gene expression in a variety of biological processes in physiological and pathological conditions [11] including autoimmune disorders [12]. They can be involved in the development of mature immune cells and in the control of their function [13]–[15]. MiRNAs are also important in the differentiation and function of the mouse intestinal epithelium [16].

In this study, we evaluated the miRNA expression pattern in the small intestine of children affected by active CD, children with CD on a gluten-free diet (GFD) and control children without CD. Our aim was to look for miRNA-based modulation of gene expression in celiac small intestine, and particularly for genes involved in cell intestinal differentiation/proliferation mechanisms.

## Results

### Clinical features of CD patients and controls

Clinical features of our CD patients and controls are reported in Table 1. Villous atrophy was subtotal or total [TIIIB: n = 3 (15%) and TIIIC: n = 17 (85%)] in all active CD patients. Only minor histological abnormalities were present in GFD patients [T0: n = 5 (56%) and TI: n = 4 (44%)] and in control patients [T0: n = 7 (64%) and TI: n = 4 (36%)].

**Table 1:** General characteristics of studied celiac patients (active CD and GFD) and control children (CTRL).

Characteristics#	Subjects		
	Active CD (n = 20)	GFD∧ (n = 9)	CTRL (n = 11)
Sex Female (%)	55	55	45
Age (Years)	4.3±1.3	7.6±2.5	6.1±1.0
Clinical presentation:			
Gastrointestinal symptoms (%)	80	22	82
Villous atrophy % (Marsh stage)‡	TIIIB 15	T0 56	T0 64
	TIIIC 85	TI 44	TI 36
Positive tTG or EMA (IgA)§	19	3	0
Familiarity for:			
CD (%)	20	22	0
Other autoimmune diseases (%)	5	11	0

# Data are expressed as percentage (%) or as mean ± standard error of the mean (SEM)

∧ At gluten free diet from at least 2 years.

‡ According to [41].

§ Only 1 active CD patient was negative for these antibodies but was positive for both AGA IgG/IgA antibodies. Borderline tTG values in 3/9 GFD patients were attributed to reported sporadic gluten ingestion.

### CD children and controls have a different miRNA expression levels in small intestine

**Figure 1:**
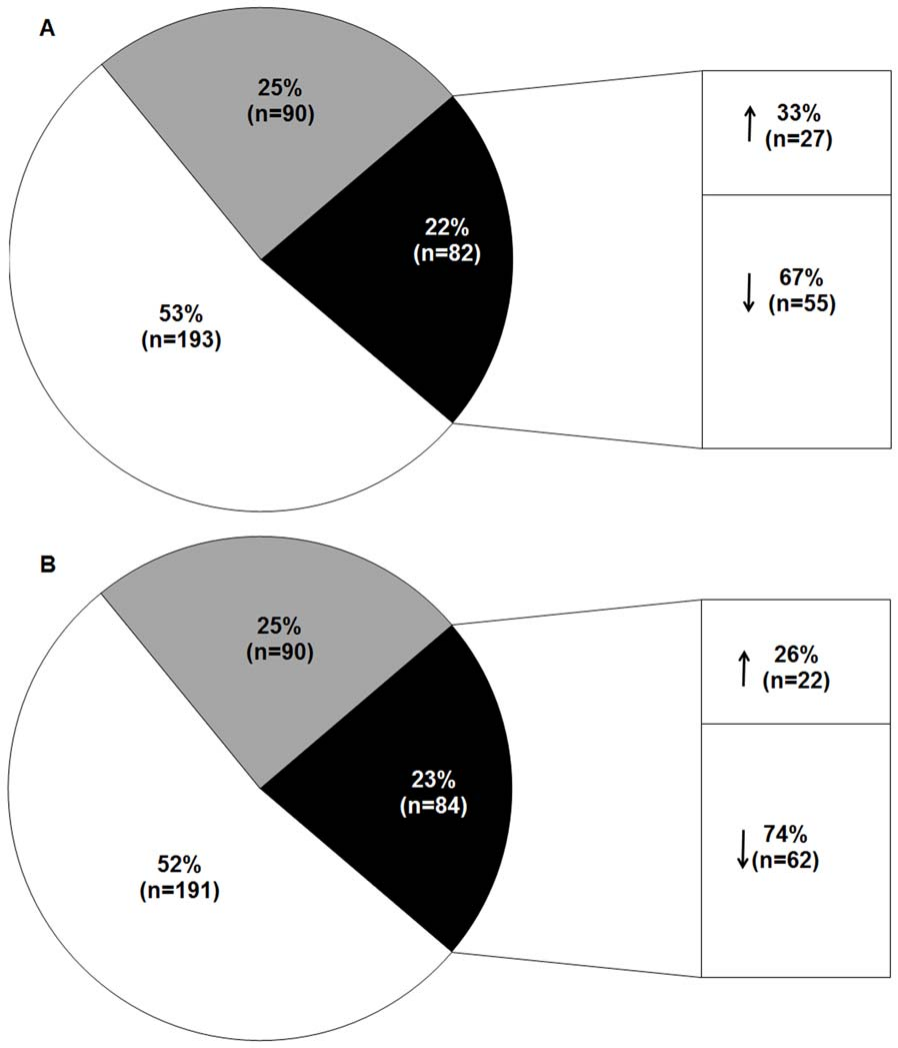
miRNA expression pattern in small intestine of CD patients. miRNA expression in the small intestine of patients with active CD (Panel A) and of CD patients on a GFD (Panel B). Data are expressed as percentage of miRNAs tested (n = 365). White areas, miRNAs whose expression levels were similar in the two CD groups and controls; gray areas, miRNAs not expressed; black areas, miRNAs whose expression levels differed between CD patients and controls (up-regulated ↑(RQ≥2.0) or down-regulated ↓(RQ≤0.5)).

Figure 1 shows the miRNA expression in the small intestine of children with active CD (panel A) and in children on a GFD (panel B). Ninety of the 365 (25%) miRNAs tested were not expressed in small intestine. Over 50% of miRNAs were expressed at similar levels in the two groups of CD compared to controls. On the contrary, the expression levels of about 20% of miRNAs (22% in active CD and 19% in GFD) differed between CD and controls. In particular, in active CD patients 27 and 55 miRNAs were expressed respectively more (RQ≥2.0) or less (RQ≤0.5) than in controls, whereas in GFD patients 22 and 49 miRNAs were expressed respectively more (RQ≥2.0) or less (RQ≤0.5) than in controls. The miRNAs that were differently expressed in the two CD groups are listed in Table S1.

### Two sets of miRNAs (one down-regulated and one up-regulated compared to controls) show similar expression levels in active and GFD CD patients, being miR-449a the highest expressed miRNA

Among the miRNAs differently expressed between CD children and controls, but with similar expression levels in active and in GFD CD, 9 were up-regulated and 21 were down-regulated (Table 2). Particularly, among the down-regulated miRNAs the set of miR-124a, miR-189, miR-299-5p and miR-379, was previously reported associated with autoimmune disorders [17]. Among the up-regulated miRNAs the miR-449a was expressed at very high levels in all active CD (55.18±16.45 mean RQ±SEM) and GFD children (15.43±7.69 mean RQ±SEM). qRT-PCR confirmed the expression levels both of miR-449a (active CD: 2.8±0.9 mean RQ±SEM) and of 2 other tested miRNAs, the down-regulated miR-124a (active CD:0.6±0.1 mean RQ±SEM) and the similar to control expressed miR-564 (active CD:1.4±0.3 mean RQ±SEM vs 1.2±0.1 at array).

**Table 2:** List of miRNAs (n = 30) differently expressed in CD patients and controls but with similar expression levels both in active CD and GFD children.

MiRNA	Active CD	GFD
**up-regulated miRNAs**		
miR-449a	55.18±16.45	15.43±7.69
miR-492	48.88±14.56	26.86±9.00
miR-644	47.80±8.80	37.53±18.85
miR-503	19.84±2.36	20.55±8.07
miR-196a	11.06±2.84	8.45±1.01
miR-504	5.54±0.83	8.02±2.86
miR-500	5.49±0.70	7.88±1.56
miR-330	3.84±0.45	2.48±0.11
miR-182	2.95±0.42	2.75±0.13
**down-regulated miRNAs**		
miR-105	0.37±0.03	0.25±0.03
miR-409-5p	0.35±0.04	0.31±0.05
miR-631	0.34±0.03	0.27±0.04
miR-659	0.33±0.03	0.30±0.05
miR-379	0.30±0.05	0.23±0.10
miR-566	0.29±0.02	0.23±0.03
miR-512-3p	0.27±0.03	0.26±0.04
miR-614	0.26±0.02	0.21±0.02
miR-380-5p	0.25±0.03	0.28±0.04
miR-135a	0.21±0.05	0.38±0.05
miR-124a	0.20±0.02	0.21±0.05
miR-600	0.19±0.02	0.22±0.06
miR-618	0.18±0.03	0.32±0.07
miR-616	0.17±0.04	0.11±0.03
miR-189	0.15±0.05	0.21±0.06
miR-576	0.15±0.04	0.40±0.10
miR-412	0.13±0.03	0.18±0.01
miR-202	0.12±0.06	0.17±0.08
miR-299-5p	0.11±0.01	0.15±0.05
miR-323	0.11±0.01	0.23±0.08
miR-219	0.10±0.01	0.27±0.08

Data are reported as RQ# levels (mean±SEM).

# RQ = 2^−delta deltaCT^ represents miRNA fold change in CD patients vs mean value obtained in control patients.

### Bioinformatic prediction of the target genes of miR-449a

Six of the 11 programs [Target Scan 5.1, PicTar, Miranda 1.9, MirTarget2 (v2.0), PITA (Catalog version 3) and RNAhybrid (v2.2)], which we used to predict putative target genes of miR-449a, identified several proteins that are present in relevant biological pathways. The biological pathways predicted to be deregulated by miR-449a and sorted in functional groups are reported in Figure S1 (http://mirecords.biolead.org/interactions.php?species=Homosapiens&mirna_acc=hsa-miR449a&targetgene_type=refseq_acc&targetgene_info=&v=yes&search_int=Search (http://www.targetscan.org/cgi-bin/targetscan/vert_50/targetscan.cgi?species=Human&gid=&mir_sc=&mir_c=&mir_nc=&mirg=hsa-miR-449a). Among putative target genes the programs identified NOTCH1, Krueppel-like factor 4 (KLF4), delta-like 1 (DLL1), lymphoid enhancer-binding factor 1 (LEF1) and numb homolog-like (NUMBL) proteins, which are all involved in the Notch pathway. As this pathway plays a relevant role in the control of intestinal cell fate in animal models we further examined the interaction of miR-449a with Notch pathway [18].

#### MiR-449a binds to the 3′ UTR of NOTCH1 and KLF4 and inhibits their expression

We verified the interaction between miR-449a and the 3′ UTR of NOTCH1 and of KLF4 using the luciferase reporter assay. In cells co-transfected with pRL-NOTCH1 vector and pre-miR-449a or with pRL-KLF4 vector, a pre-miR-449a concentration of 100 nmol/L was sufficient to significantly reduce (respectively, p = 0.001 and p = 0.002) *Renilla* luciferase activity versus control values after 48 h (Figure S2A and S2B).This finding confirms the interaction between miR-449a and the 3′ UTR of both NOTCH1 and KLF4.

The direct interaction between miR-449a and the 3′UTRs of both NOTCH1 and KLF4 was further confirmed after mutating the putative target sites in 3′UTR of the two genes (See Materials and Methods S1).

### NOTCH1 and HES1 mRNAs are expressed in the small intestine of CD patients

NOTCH1 and HES1 mRNA levels, tested by qRT-PCR, were expressed in the small intestine of active CD patients (RQ±SEM: 3.4±1.3 and 1.4±0.2, respectively *vs* controls) and of GFD patients (RQ±SEM: 6.5±4.7 and 0.7±0.1, respectively vs controls).

### NOTCH1 and HES1 proteins are underexpressed in the small intestine of CD patients

**Figure 2:**
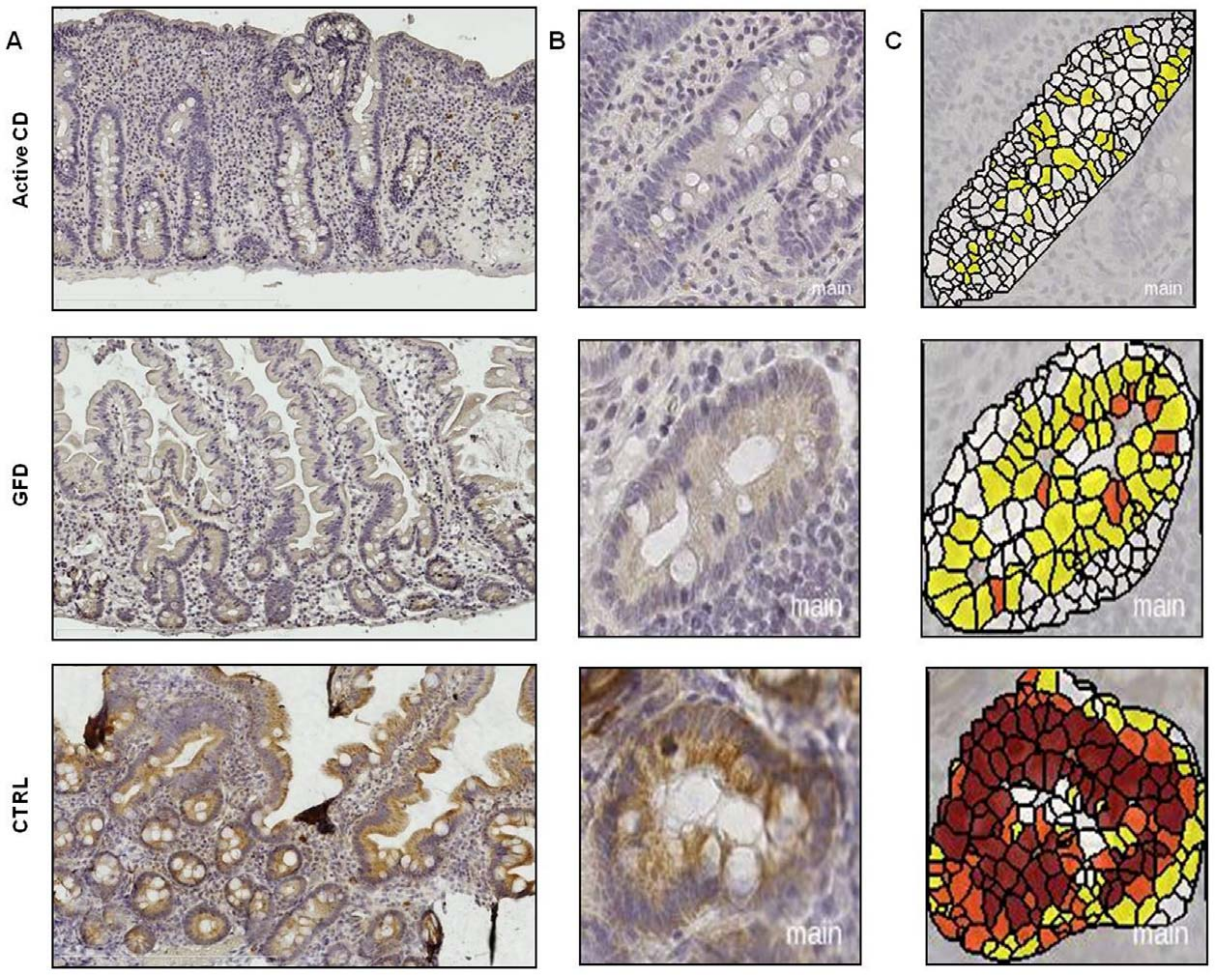
Decreased expression of NOTCH1 in small intestine of CD patients compared with controls. An example of NOTCH1 immunohistochemistry in small intestine. **A.** Low magnification picture of the intestinal sections (Original magnification 10×). **B.** Intestinal crypts (Original magnification 40×). Note the homogeneous distribution of NOTCH1 in crypts and along the villi in control sample, whereas in active CD and GFD samples the signals were prevalently detected in the crypts. Higher levels of NOTCH1 were detected in the intestinal crypts of controls than in crypts of active and GFD CD samples. **C.** Images converted for automated analysis (white: unstained cells, yellow/orange: low/moderately stained cells, brown: intensely stained cells). These results indicate that NOTCH1 is less expressed in the small intestine of active and GFD CD samples compared with controls. (CTRL: controls; GFD: gluten free diet; CD: celiac disease).

We next investigated the protein expression of NOTCH1 and of HES1, which is a well known target gene of the Notch receptor family, in small intestinal biopsies from CD patients and controls. Figure 2 shows the results obtained for NOTCH1. NOTCH1 was homogeneously distributed in the intestinal villi and crypts of controls and higher expressed in crypts of controls than in crypts of active and GFD CD patients (panel A, B).

**Figure 3:**
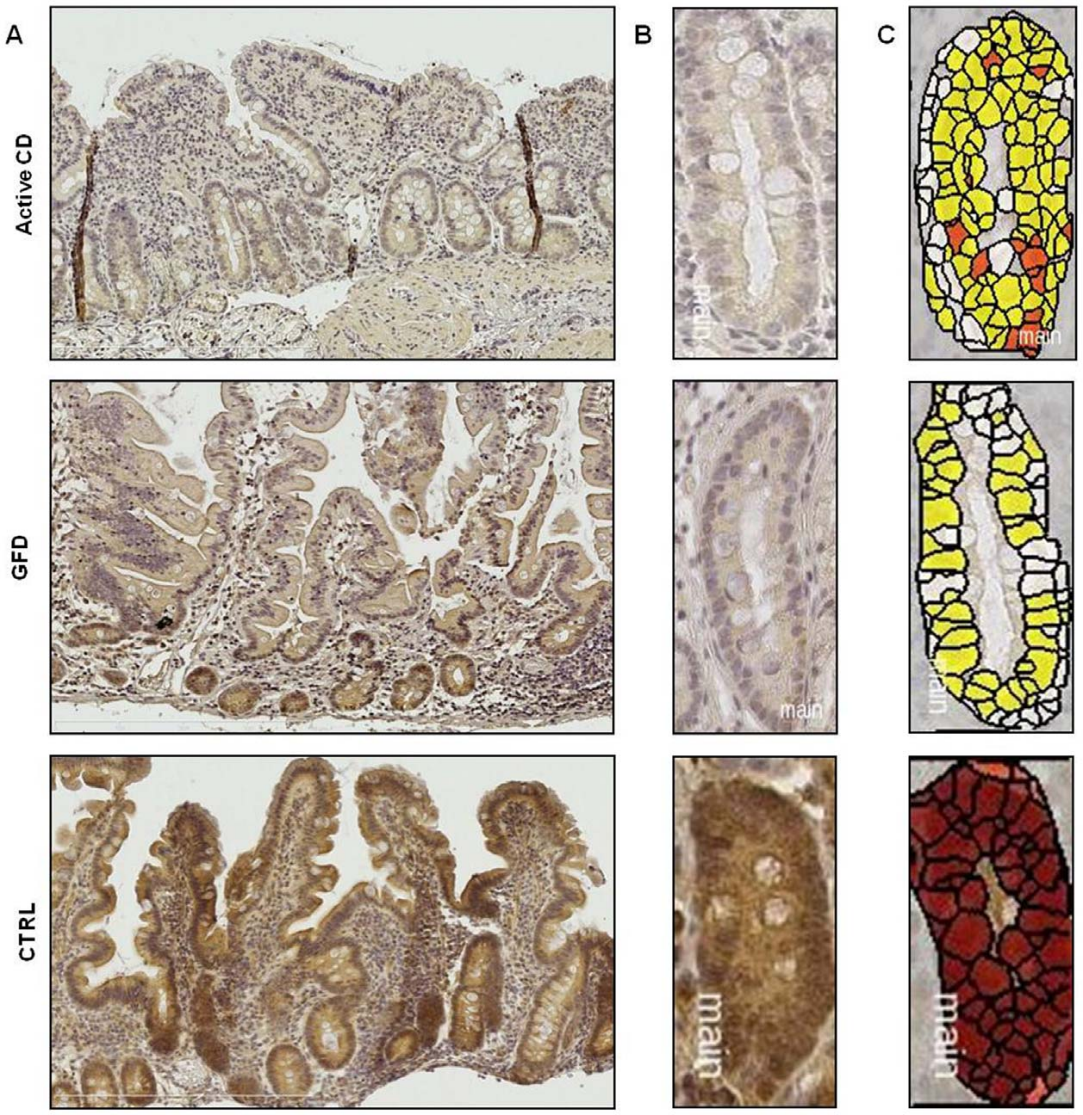
Decreased expression of HES1 in small intestine of CD patients compared with controls. An example of HES1 immunohistochemistry in small intestine. **A.** Low magnification picture of the examined intestinal sections (Original magnification 10×). **B.** Intestinal crypts (Original magnification 40×). Note the homogeneous distribution of HES1 in crypts and along the villi in control sample, whereas the signals were prevalently detected in the crypts of active CD and GFD samples. Higher levels of HES1 were detected in the intestinal crypts of controls than in crypts of active and GFD CD samples. **C.** Images converted for automated analysis (white: unstained cells, yellow/orange: low/moderately stained cells, brown: intensely stained cells). These results indicate that HES1 is less expressed in the small intestine of active and GFD CD samples compared with controls. (CTRL: controls; GFD: gluten free diet; CD: celiac disease).

In Figure 2 (panel C) are also the images converted for automated analysis (white: unstained cells, yellow/orange: low/moderately stained cells, brown: intensely stained cells). Significantly more intensely stained and less unstained cells (p = 0.02) were detected in controls than in the two groups of CD patients, whereas no statistical significant difference was observed between the two CD groups (Figure S3, panel A and Figure S4). These results indicate that NOTCH1 is less expressed in the small intestine of active and GFD CD patients than in controls. Figure 3 shows the results obtained for HES1. HES1 was homogeneously distributed in the intestinal villi and crypts of controls and higher expressed in crypts of controls than in crypts of active and GFD CD patients (panels A and B). In Figure 3 (panel C) are also the images converted for automated analysis (white: unstained cells, yellow/orange: low/moderately stained cells, brown: intensely stained cells). Significantly more intensely stained cells were detected in controls than in CD patients (p = 0.02) and significantly less unstained cells were detected in controls than in active CD patients (p = 0.03), whereas no statistical significant difference was observed between the two CD groups (Figure S3, panel B and Figure S5). These results indicate that HES1 is less expressed in the small intestine of active and GFD CD patients with respect to controls. The above findings confirm that NOTCH1 signaling is reduced in patients affected by CD.

### KLF4 protein is reduced and the number of goblet cells is significantly lower in the small intestine of CD patients versus controls

**Figure 4:**
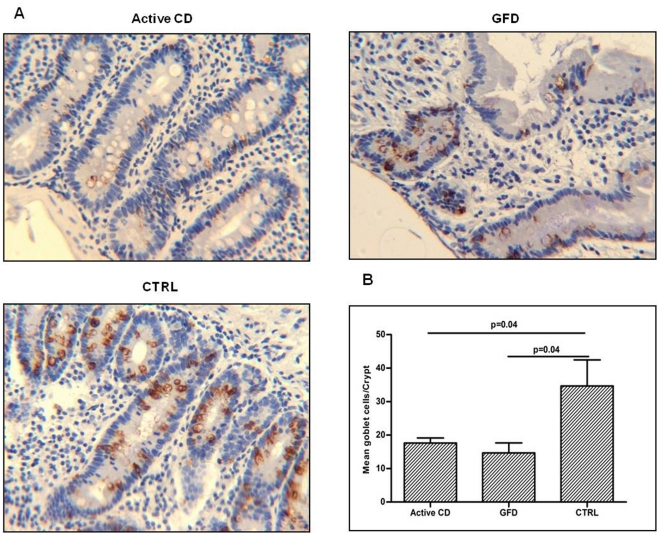
Decreased expression of MUC2 in small intestine of CD patients compared with controls. Immunohistochemistry of goblet cells in small intestine. **A.** An example of staining for MUC-2 shows fewer MUC-2 stained cells in active and in GFD CD samples than in controls. (Original magnification 20×). **B.** MUC2 stained cells evaluated in CD patients (6 active CD and 6 GFD patients) and in controls (n = 4). Data are expressed as mean of the number of goblet cells/crypt measured in 10 crypts/children. Significantly fewer stained cells were detected in active and GFD CD samples than in controls (p = 0.04). These results indicate that MUC2 is less expressed in small intestine of active and GFD CD patients compared with controls. (CTRL: controls; GFD: gluten free diet; CD: celiac disease).

We also investigated the protein expression of KLF4, another selected target gene of miR449a, in small intestinal villi from GFD patients and controls, lacking the villous architecture in active CD patients. We found that the levels of this protein (mean±SEM) were significantly lower in villi from GFD patients vs controls, respectively 29.0±5.0 vs 79.0±3.0 (p<0.0001) (Figure S6, panel A). Since KLF4 negatively regulates cellular proliferation, we examined the effect of inhibition of KLF4 on the proliferation of intestinal crypts with the proliferation marker Ki67. The results show that the number of Ki67 positive cells is higher in the crypts of CD patients than in controls (Figure S6, panel B). Because KLF4 is also involved in the differentiation and maturation of secretory goblet cells we examined these cells by immunohistochemistry and using anti-MUC-2 antibodies. We detected statistically fewer MUC-2-stained cells (mean±SEM) in the crypts of active CD patients (18.0±1.6) and GFD patients (15.0±3.0) than in controls (35.0±7.7) (p = 0.04) (Figure 4A and B). Moreover, there were fewer goblet cells in the villi of GFD patients (7.0±1.8) than in controls (20.0±4.9) (p = 0.04) (data not shown). This finding demonstrates that the differentiation of the secretory goblet cells is impaired in small intestine of CD patients.

### Expression of beta-catenin

Because NOTCH1 and also KLF4 interact with the WNT pathway to influence the intestinal stem cell fate, we investigated the WNT pathway using beta-catenin antibodies. By counting the beta-catenin positive nuclei/crypt for each patient we observed higher even if not statistical significant mean percentage beta-catenin positive nuclei/crypt in active CD and GFD patients than in controls, respectively 57.0±11.5 and 37.0±4.6 *vs* 27.0±4.6 (Figure S7). This finding suggests that cellular proliferation is increased in the small intestine of CD patients.

## Discussion

A very recent study established the importance of miRNAs in the differentiation and function of the mouse intestinal epithelium [16], whereas there are no data about miRNAs expression in human CD small intestine. Our study reveals that the expression of about 20% of miRNAs tested in the small intestine differed among CD and control children irrespective of whether the disease was active or not. Particularly, the miR-449a showed the highest expression level in CD patients than in controls. The miR-449 (a and b) cluster is embedded into an intronic sequence of the mRNA-encoding gene *CDC20B* on Chr 5q11.2 [17]. MiR-449a seems to be regulated through activation of its host gene, *CDC20B*, and both were induced by the cell cycle regulator E2F1 [19]. The mature miR-449a sequence is evolutionarily conserved across a variety of species (monkey, horse, rodents, and dogs) and therefore it probably exerts an important function [20]. The bioinformatics search for putative target genes of miR-449a revealed about one hundred proteins, among these several belonged to the Notch pathway, i.e., NOTCH1, KLF4 (a NOTCH1 transcription factor) [21], DLL1, LEF1 and NUMBL. Our strategy to choose NOTCH1 gene among the other putative miRNA-target genes was based on many studies highlighting that cellular formation of the villi in small and large intestine is affected by signaling pathways such as Notch, Wnt and BMP [22]–[25]. Notably, deregulation of the intestinal epithelial formation has been reported in several intestinal diseases such as Crohn, ulcerative colitis and colon cancer [26]. Further, NOTCH1 and KLF4 genes are both involved in the control of mouse intestinal epithelial homeostasis [18], [27]. In fact, in mouse intestine, also in cooperation with WNT signals, NOTCH1 guides cell proliferation and differentiation [18] and KLF4 inhibition by NOTCH1 or KLF4 deletion was shown to reduce the differentiation and maturation of goblet cells [21], [27]–[29]. The Notch family is constituted by single transmembrane receptors that, in mammals, after interaction with ligands (DLL1,3,4 and Jagged 1–2) undergo proteolytic cleavage and finally translocate into the nucleus where they activate target gene transcription [30].

After confirmed the interaction between miR-449a and both NOTCH1 and KLF4 mRNA, we measured the NOTCH1 and KLF4 protein levels in small intestinal biopsies of CD children. NOTCH1-positive cells were significantly fewer in biopsies from active and GFD CD patients versus controls. Similar results were also obtained for HES1, a target gene of NOTCH1 [31]. Globally, these data indicate that the NOTCH1 pathway is deregulated in intestinal epithelium of CD children, irrespective of whether the disease is active or not, and that this alteration could be related to the very high miR-449a expression. Accordingly, in a very recent report miR449 by repressing the Delta/Notch pathway was elegantly shown to control the human airway epithelium and vertebrate multilciliogenesis [32]. In our patients we also observed fewer KLF4-positive cells in small intestinal villi from GFD patients than in controls, and, moreover, Ki67 signals were higher in crypts from CD patients versus controls. These two results are in agreement with data very recently reported in a mouse KLF4^DELTAIS^ model [27] and indicate a higher proliferation rate in our CD patients in the presence of reduced KLF4 expression [27]. In parallel the number of goblet cells was significantly lower in the two CD groups than in control children. Ciacci et al [33] previously reported fewer goblet cells/mm^2^ in untreated (29.1) and in treated CD patients (42.2) than in controls (50.5), although the differences were statistically significant only in untreated patients (p<0.02).

The WNT pathway in the small intestine of our CD patients, evaluated based on beta-catenin expression level, did not differ from that of control children. This result is in agreement with the western blot data reported by Ciccocioppo et al [34] and by Juuti-Uusitalo et al [7]. However, we observed a more evident nuclear localization of beta-catenin, albeit not statistically significant, in the small intestinal crypts from our active and GFD CD patients than in controls, which suggests activation of the WNT pathway. The latter finding is in agreement with a previous study of human CD [7] and with the increased mRNA levels of the genes in the WNT pathway, including beta-catenin, observed in the KLF4^DELTAIS^ mouse [27]. Globally, our data support increased cellular proliferation in the small intestinal epithelium in CD patients. As it is well known, active CD is characterized by an inversion of the differentiation/proliferation program of the intestine with a reduction in the differentiated compartment, up to complete villous atrophy, and an increase of the proliferative compartment, with crypt hyperplasia [7], [8]. Furthermore, although GFD intestinal mucosa is characterized by an apparently normal mucosal architecture, it can also be associated with increased crypt cell proliferation (Barone M. V. et al., personal communication). Our data are in contrast with those obtained in mouse models, in which NOTCH1 activation resulted in a reduction of goblet cells consequent to HES-1 dependent repression of Math1 (intestinal secretory cell differentiation factor) [18] and in which NOTCH1 inhibited the expression of KLF4 [35]. However, our data are in agreement with a recent report of increased proliferation, reduced differentiation and goblet cells maturation associated with down-regulation of the expression of components of the Notch pathway (HES1, DLL1, JAG1) in the small intestine of the KLF4^DELTAIS^ mouse [27]. The authors for the latter article hypothesized that KLF4 was involved in a feed-back loop by positively regulating Notch signaling. Our results are suggestive that an altered NOTCH1 and KLF4 expression could lead to the reduction of goblet cells in the small intestine of CD patients. The maintenance of a correct number of functional goblet cells is required for the homeostasis of the intestinal mucosal environment, and deficiencies in the mucin composition renders the mucosa more susceptible to damaging agents in the lumen [36]–[38]. In fact, loss of goblet cell function leads to spontaneous colitis in mice [39]. Moreover, an altered mucous layer and increased rod-shaped bacteria and interferon-gamma mRNA levels were found in intestine from CD patients [40]. Based on these experimental data, we suggest that the mucus layer in our CD children could be altered so deranging the protective function of the mucosal barrier that interfaces with the environment. In our study, the observed small intestine alterations are not related to inflammation; in fact, they occurred in both the active CD and GFD patients. The major criticism in our work is the gap between the results of the miRNA array with NOTCH1 gene in *a vivo* system, however the lack of a celiac animal model at moment, prevent us from this further validation of our data. Nevertheless, our first description of miRNA pattern in celiac disease and of the correlation of miRNA 449a over expression with NOTCH pathway could pave the way for further research in this field. However, our choice to study Notch pathway doesn't exclude that other relevant biological pathways in addition to it could be miRNA-deregulated in the celiac intestine. Further deeper investigation are necessary to test this hypothesis.

In conclusion we first demonstrate a miRNA mediated gene regulation in small intestine of CD patients. We also highlighted a reduced NOTCH1 pathway in our patients, irrespective of whether the disease was active or not. We suggest that NOTCH pathway could be constitutively altered in the celiac small intestine and could drive the increased proliferation and the decreased differentiation of intestinal cells towards the secretory goblet cell lineage.

## Materials and Methods

### Ethics approval

The study was conducted according to the Helsinki II declaration and it was approved by the Ethics Committee of the School of Medicine Federico II, Naples, Italy.

Written informed consent was obtained from the parent/guardian of all children involved in our study before their enrollment.

### Patients and controls

Forty-four children were recruited, in a two months period, among patients attending the Department of Paediatrics of the University of Naples Federico II where the European Laboratory for the Investigation of Food-Induced Diseases (ELFID) is also present. In our center about 40 biopsies are monthly performed and about 50% of them are usually indicative of CD. Twenty/44 children were diagnosed celiacs according to the criteria established by the European Society for Paediatric Gastroenterology, Hepatology and Nutrition (ESPGHAN) [41]; the CD was excluded based on both absence of CD antibodies and slight or no abnormalities in the mucosal architecture in 15/44 children. In four of these latter children (4/15) the final diagnoses were IgA deficiency (2 cases), De George syndrome (1 case) and autoimmune thyroiditis (1 case), these subjects were excluded from the study to avoid potentially confounding diseases. In the other 11/15 CD-negative children the final diagnoses were: *Helicobacter pylori* infection, recurrent vomiting, food refusal or reflux esophagitis, they were our enrolled controls. Nine out 44 children were CD patients on gluten free diet for at least 2 years undergoing CD follow-up in the same study period of the active CD patients and controls. There was no statistically significant difference in mean age at diagnosis among the groups evaluated (4.3±1.3 years old in active CD subjects, 7.6±2.5 in GFD subjects, and 6.1±1.0 in controls [mean±SEM]). About 50% of each group was girls. >From all participants, we collected a fasting serum sample, a blood sample with EDTA, and a small intestine biopsy sample.

### Biochemical parameters

Anti-Endomysium IgA were detected by indirect immunofluorescence on rhesus monkey esophagus substrate (Eurospital, Trieste, Italy); tTG IgA, anti-gliadin (AGA) IgA/IgG were analyzed by ELISA with human recombinant tTG as antigen (DIA Medix Corp., Miami, FL, USA).

### Histopathological analysis

Architectural abnormalities were classified according to the modified Marsh classification: normal mucosa (T0), intraepithelial lymphocytosis (TI), intraepithelial lymphocytosis and crypt hyperplasia (TII), intraepithelial lymphocytosis, crypt hyperplasia and villous atrophy (partial TIIIA, subtotal TIIIB, total TIIIC) [42].

### DNA and RNA extraction

Genomic DNA was extracted using the Nucleon BACC 2 kit (Amersham Biosciences Europe, Milan, Italy). Total RNA, including miRNAs, was extracted from small intestinal biopsy samples using the Mirvana extraction kit (Applied Biosystems, Foster City, CA, USA).

### HLA typing

DQ2/DQ8 HLA CD-associated molecules were identified by using primers and the PCR conditions of a commercial kit (BAG Health care GmbH, Lich, Germany), which allows to identify the HLA-alleles coding DQ2/DQ8 molecules.

### MiRNAs evaluation

TaqMan low density arrays (TLDA), micro fluidic cards were used to detect and quantify mature miRNAs (Applied Biosystems' 7900HT) according to manufacturer's instructions (see Materials and Methods S1 for details). We considered differently expressed in CD vs controls, the miRNAs whose mean RQ levels were ≤0.5 (down-regulated) or ≥2.0 (up-regulated).

### Bioinformatic approach

The prediction of putative target genes of miRNAs was determined using miRecords (http://mirecords.biolead.org/), which is an integration of 11 established miRNA target prediction programs. The lists of target genes that were predicted by two or more programs were then combined and analyzed using the Gene Ontology Tree Machine (GOTM) (http://bioinfo.vanderbilt.edu/gotm/) and KEGG database (http://www.genome.ad.jp/kegg/). Finally, we identified the biological pathways that contained at least two up- or down-regulated genes with a statistically significant probability (p<0.01).

### Quantitative real-time polymerase chain reaction (qRT-PCR) of miRNAs and mRNAs

The levels of a group of deregulated miRNAs (up-regulated miR-449a, down-regulated miR-124a, and similar to controls expressed miR-564) were also evaluated with TaqMan miRNA assays (Applied Biosystems) to validate the array results.

mRNA expression levels of neurogenic locus notch homolog protein 1 (NOTCH1) and of hairy and enhancer of split 1 (HES1) were measured in small intestinal tissues by qRT-PCR using single TaqMan mRNA assays (Applied Biosystems) according to the manufacturer's instructions and using the housekeeping gene beta-actin as control. Reverse transcription reactions were performed with the High Capacity cDNA Reverse Transcription Kit (Applied Biosystems). The expression levels of miRNAs and mRNAs were quantified using the ABI Prism 7900HT Sequence Detection System 2.3 software.

### Transfection and inhibition experiments

The oligonucleotides, plasmids (pGL3-control, pRL-NOTCH1-encoding, pRL-KLF4-encoding and mutated pR-KLF4-encoding, firefly luciferase and Renilla luciferase, respectively) and human embryonic kidney cell lines (HEK293 cell line, ATCC number CRL-1573, supplied by the Centre for Applied Microbiology and Research, Salisbury, Wiltshire, UK) used for cell transfection experiments are described in detail in the online Materials and Methods S1.

Forty-eight hours after transfection, we measured firefly and *Renilla* luciferase activities using a dual luciferase assay according to the manufacturer's instructions (Promega, Naples, Italy).

### Protein evaluation by immunohistochemistry

Given the small amount of sample available for each patient (1–2 mg of intestinal tissue/patient) we tested the expression of selected proteins by immunohistochemistry instead of by western blotting. The NOTCH1, HES1, MUC-2, KLF4, Ki67 and beta-catenin proteins were identified on formalin-fixed paraffin-embedded small intestinal tissue blocks in CD patients and in controls. We randomly selected six active CD, six GFD and four controls (see Materials and Methods S1 for details). We also tested the specificity of our NOTCH1 and HES1 signals evaluating two different human tissue samples where it is known NOTCH1 and HES1 be present or absent respectively, that are colon cancer and endothelial wall (Figure S8).

### Scanning and automated image analysis of NOTCH1 and HES1

To increase precision, we automated the quantification of the immunohistochemical signals. Sections of the small intestine were scanned with the NanoZoomer 2.0 system (Hamamatsu, Japan), equipped with a 20×, 0.7 Numerical Aperture Plan-Apochromat lens, using a lens of 0.23 mm pixel size. The compressed jpeg files were transferred to the Definiens Analyst LS5.0 system (Definiens AG, Germany) that counted the NOTCH1, HES1 and beta-catenin -positive and -negative cells and quantified the staining signal (see materials and methods S1 for Definiens Analyst software details). The Definiens Analyst software (Definiens AG, Germany) is based on cognition network technology that is a semantic network of objects and their mutual relationships. Two rule sets, using cognition network language, were specifically written for this evaluation to automatically detect and measure the small intestinal area and to count positive and negative crypt cells. The signal was classified as intensely stained, low/moderate stained and unstained. Thus, both the percentage and intensity of labeled cells were taken into account. The detection and exclusion of areas not belonging to crypt were visually checked for all image files. Ten crypts/patient were counted.

### Immunohistochemical analysis of MUC-2, KLF4 and beta-catenin

Because the MUC-2 staining of goblet cells was patchy, we picked ten crypts from each slide and manually counted the number of goblet cells stained in each crypt. We also evaluated MUC-2 staining of villi, when possible, i.e., in GFD patients and controls. We also evaluated KLF4-positive villi (in GFD patients) and both beta-catenin- and Ki67-positive nuclei/crypt in each subject. Two independent observers evaluated the immunohistochemical slides.

### Statistics

All variables were expressed as mean±standard error of the mean (SEM). Student t's test and ANOVA were used to compare group means and p values<0.05 were considered significant. Statistically significant (p<0.01) miRNA-regulated pathways were selected by the GOTM program.

## Supporting Information

Materials and Methods S1 (DOC)

Figure S1 **Bioinformatics analysis of miR-449a putative target genes.** miR-449a putative target genes with most favorable context score, selected by bioinformatics, were sorted into pathways using GOTM (http://bioinfo.vanderbilt.edu/webgestalt/) and then combined into functional groups. (http://mirecords.biolead.org/interactions.php?species=Homosapiens&mirna_acc=hsa-miR-449a&targetgene_type=refseq_acc&targetgene_info=&v=yes&search_int=Search) (http://www.targetscan.org/cgibin/targetscan/vert_50/targetscan.cgi?species=Human&gid=&mir_sc=&mir_c=&mir_nc=&mirg=hsa-miR-449a). In each functional group are reported the genes belonging to NOTCH pathway/total gene number.
(TIF)

Figure S2 **The luciferase assay confirms that miR-449a inhibits the expression of NOTCH1 and KLF4.** In HEK293 cells co-transfected or with pRL-NOTCH1 vector (panel A) or with pRL-KLF4 vector (panel B), a pre-miR-449a concentration of 100 nmol/L was sufficient to significantly reduce (respectively, p = 0.001 and p = 0.002) *Renilla* luciferase activity versus control values. No inhibition of the *Renilla* luciferase expression was observed in mutant 3′UTR of KLF4-mRNA with miR-449a, so confirming the miR-449a/3′UTR KLF4-mRNA direct interaction (panel B). We didn't verify the interaction miR-449a/3′UTR NOTCH1 being this latter recently validated by Marcet B et al [32].
(TIF)

Figure S3 **Automated Counts of NOTCH1 and HES1 stained/unstained cells.** **A.** Automated counts of NOTCH1 stained/unstained cells (reported in Figure 2) in small intestine from CD patients (6 active CD and 6 GFD patients) and from controls (n = 4). Data are expressed as mean percent of intensely stained, low-moderately stained and unstained cells of the total intraepithelial cells (IECs) counted in ten crypts. The numbers of intensely stained and unstained cells were significantly (p = 0.02) higher and lower, respectively, in CTRL than in active CD and in GFD patients. **B.** Automated counts of HES1 stained/unstained cells (reported in Figure 3) in small intestine from CD patients (6 active CD and 6 GFD patients) and from controls (n = 4). Data are expressed as mean percent of intensely stained, low-moderately stained and unstained cells of the total intraepithelial cells (IECs) counted in ten crypts. The number of intensely stained cells was significantly higher in controls versus CD and GFD patients (p = 0.02) and the number of unstained cells was significantly lower in CTRL than in active CD patients (p = 0.03). (CTRL: controls; GFD: gluten free diet; CD: celiac disease).
(TIF)

Figure S4 **Other examples of NOTCH1 immunohistochemistry in CD patients.** Examples of NOTCH1 immunohistochemistry in 4 CD patients (2 active CD: TIII Marsh stage and 2 GFD: TI and T0 Marsh stage) and 2 controls (T0 Marsh stage). The images show that the low expression levels of NOTCH1 in intestinal mucosa from CD patients were always present from TIII to T0 Marsh stage. (CTRL: controls; GFD: gluten free diet; CD: celiac disease).
(TIF)

Figure S5 **Other examples of HES1 immunohistochemistry in CD patients.** Examples of HES1 immunohistochemistry in 4 CD patients (2 active CD: TIII Marsh stage and 2 GFD: TI and T0 Marsh stage) and 2 controls (T0 Marsh stage). The images show that the low expression levels of HES1 in intestinal mucosa from CD patients were always present from TIII to T0 Marsh stage. (CTRL: controls; GFD: gluten free diet; CD: celiac disease).
(TIF)

Figure S6 **Decreased KLF4 and increased Ki67 expression in small intestine from CD patients compared with controls.** **A.** KLF4 staining of small intestinal villi in GFD patients and Controls (Original magnification 20×). A statistically significant reduced KLF4-positive cells/villi were counted in GFD patients than in controls, respectively 29.0±5.0 vs 79.0±3.0 (mean±SEM) (p<0.0001). **B.** Increased Ki67 signal is present in small intestinal crypts of active CD, GFD patients than in controls (Original magnification 20×). (CTRL: controls; GFD: gluten free diet; CD: celiac disease).
(TIF)

Figure S7 **Increased expression of beta-catenin in small intestine from CD patients compared with controls.** Immunostaining with beta-catenin in small intestinal crypts from active CD, GFD and controls. We counted the beta-catenin labeled nuclei. Similar counts of beta-catenin labelled nuclei were detected in the crypts of the small intestine in all groups. However, higher even if not statistical significant mean percentage counts (beta-catenin positive nuclei/crypt) were obtained in active CD and GFD than in controls, respectively 57.0±11.5 and 37.0±4.6 vs 27.0±4.6 (Original magnification 63×). (CTRL: controls; GFD: gluten free diet; CD: celiac disease).
(TIF)

Figure S8 **Specificity of NOTCH1 and HES1 signals by immunohistochemistry.** Specificity controls of NOTCH1 and HES1 antibodies. Positive NOTCH1 (**A**) and HES1 (**B**) immunostaining signals obtained in human colon cancer and negative NOTCH1 (**C**) and HES1 (**D**) immunostaining signals obtained in human endothelial wall.
(TIF)

Table S1 **MiRNAs differently expressed in active and GFD CD patients.**
(DOC)

## References

[1] Nisticò L, Fagnani C, Coto I, Percopo S, Cotichini R (2006). Concordance, disease progression, and heritability of coeliac disease in Italian twins.. Gut.

[2] Greco L, Romino R, Coto I, Di Cosmo N, Percopo S (2002). The first large population based twin study of coeliac disease.. Gut.

[3] Schuppan D, Junker Y, Barisani D (2009). Celiac disease: from pathogenesis to novel therapies.. Gastroenterology.

[4] Hunt KA, van Hell DA (2009). Recent advances in celiac disease genetics.. Gut.

[5] Sacchetti L, Calcagno G, Ferrajolo A, Sarrantonio C, Troncone R (1998). Discrimination between celiac and other gastrointestinal disorders in childhood by rapid human lymphocyte antigen typing.. Clin Chem.

[6] Van Heel DA, Franke L, Hunt KA, Gwilliam R, Zhernakova A (2007). A genome-wide association study for celiac disease identifies risk variants in the region harboring IL2 and IL21.. Nat Genet.

[7] Juuti-Uusitalo K, Mäki M, Kainulainen H, Isola J, Kaukinen K (2007). Gluten affects epithelial differentiation-associated genes in small intestinal mucosa of coeliac patients.. Clin Exp Immunol.

[8] Diosdado B, Wapenaar MC, Franke L, Duran KJ, Goerres MJ (2004). A microarray screen for novel candidate genes in coeliac disease pathogenesis.. Gut.

[9] Inui M, Martello G, Piccolo S (2010). MicroRNA control of signal transduction.. Nature review.

[10] Lee RC, Feinbaum RL, Ambros V (1993). The C. elegans heterochronic gene lin-4 encodes small RNAs with antisense complementarity to lin-14.. Cell.

[11] Pauley KM, Chan EKL (2008). MicroRNAs and their emerging roles in immunology.. Ann NY Acad Sci.

[12] Pauley KM, Cha S, Chan EKL (2009). MicroRNA in autoimmunity and autoimmune diseases.. J Autoimmun.

[13] Neilson JR, Zheng GXY, Burge CB, Sharp PA (2007). Dynamic regulation of miRNA expression in ordered stages of cellular development.. Genes Dev.

[14] Wu H, Neilson JR, Kumar P, Manocha M, Shankar P (2007). miRNA profiling of naïve, effector and memory CD8 T cells.. PLoS One.

[15] Zhou B, Wang S, Mayr C, Bartel DP, Lodish HF (2007). miR-150, a microRNA expressed in mature B and T cells, blocks early B cell development when expressed prematurely.. Proc Natl Acad Sci U S A.

[16] McKenna LB, Schug J, Vourekas A, McKenna JB, Bramswig NC (2010). MicroRNAs control intestinal epithelial differentiation, architecture, and barrier function.. Gastroenterology.

[17] Iborra M, Bernuzzi F, Invernizzi P, Danese S (2010). MicroRNAs in autoimmunity and inflammatory bowel disease: Crucial regulators in immune response.. Autoimmun Rev.

[18] Fre S, Pallavi SK, Huyghe M, Laé M, Janssen KP (2009). Notch and Wnt signals cooperatively control cell proliferation and tumorigenesis in the intestine.. Proc Natl Acad Sci.

[19] Lizé M, Pilarski S, Dobbelstein M (2010). E2F1-inducible microRNA 449a/b suppresses cell proliferation and promotes apoptosis.. Cell Death Differ.

[20] Noonan EJ, Place RF, Pookot D, Basak S, Whitson JM (2009). miR-449a targets HDAC-1 and induces growth arrest in prostate cancer.. Oncogene.

[21] McConnell BB, Ghaleb AM, Nandan MO, Yang VW (2007). The diverse functions of Krüppel-like factors 4 and 5 in epithelial biology and pathobiology.. Bioessays.

[22] Clevers H (2006). Wnt/beta-catenin signaling in development and disease.. Cell.

[23] Fre S, Huyghe M, Mourikis P, Robine S, Louvard D (2005). Notch signals control the fate of immature progenitor cells in the intestine.. Nature.

[24] Oshima S, Nakamura T, Namiki S, Okada E, Tsuchiya K (2004). Interferon regulatory factor 1 (IRF-1) and IRF-2 distinctively up-regulate gene expression and production of interleukin-7 in human intestinal epithelial cells.. Mol Cell Biol.

[25] Crosnier C, Stamataki D, Lewis J (2006). Organizing cell renewal in the intestine: stem cells, signals and combinatorial control.. Nat Rev Genet.

[26] Okamoto R, Tsuchiya K, Nemoto Y, Akiyama J, Nakamura T (2009). Requirement of Notch activation during regeneration of the intestinal epithelia.. Am J Physiol Gastrointest Liver Physiol.

[27] Ghaleb AM, McConnell BB, Kaestner KH, Yang VW (2011). Altered intestinal epithelial homeostasis in mice with intestine-specific deletion of the Krüppel-like factor 4 gene.. Developmental Biology.

[28] Zheng H, Pritchard DM, Yang X, Bennett E, Liu G (2009). KLF4 gene expression is inhibited by the notch signaling pathway that controls goblet cell differentiation in mouse gastrointestinal tract.. Am J Physiol Gastrointest Liver Physiol.

[29] Katz JP, Perreault N, Goldstein BG, Lee CS, Labosky PA (2002). The zinc-finger transcription factor Klf4 is required for terminal differentiation of goblet cells in the colon.. Development.

[30] Kopan R, Ilagan MXG (2009). The canonical Notch signaling pathway: unfolding the activation mechanism.. Cell.

[31] Yin L, Velazquez OC, Liu ZJ (2010). Notch signaling: emerging molecular targets for cancer therapy.. Biochem Pharmacol.

[32] Marcet B, Chevalier B, Luxardi G, Curaux C, Zaragosi LE (2011). Control of vertebrate multiciliogenesis by miR-449 through direct repression of the Delta/Notch pathway.. Nat Cell Biol.

[33] Ciacci C, Di Vizio D, Seth R, Insabato G, Mazzacca G (2002). Selective reduction of intestinal trefoil factor in untreated coeliac disease.. Clin Exp Immunol.

[34] Ciccocioppo R, Finamore A, Ara C, Di Sabatino A, Mengheri E (2006). Altered Expression, Localization, and Phosphorylation of Epithelial Junctional Proteins in Celiac Disease.. Am J Clin Pathol.

[35] Ghaleb AM, Aggarwal G, Bialkowska AB, Nandan MO, Yang VW (2008). Notch inhibits expression of the Krüppel-like factor 4 tumor suppressor in the intestinal epithelium.. Mol Cancer Res.

[36] McAuley JL, Linden SK, Png CW, King RM, Pennington HL (2007). MUC1 cell surface mucin is a critical element of the mucosal barrier to infection.. J Clin Invest.

[37] Corfield AP, Myerscough N, Longman R, Sylvester P, Arul S (2000). Mucins and mucosal protection in the gastrointestinal tract: new prospects for mucins in the pathology of gastrointestinal disease.. Gut.

[38] Festen EA, Szperl AM, Weersma RK, Wijmenga C, Wapenaar MC (2009). Inflammatory bowel disease and celiac disease: overlaps in the pathology and genetics, and their potential drug targets.. Endocr Metab Immune Disord Drug Targets.

[39] Van der Sluis M, De Koning BA, De Bruijn AC, Velcich A, Meijerink JP (2006). Muc2-deficient mice spontaneously develop colitis, indicating that MUC2 is critical for colonic protection.. Gastroenterology.

[40] Forsberg G, Fahlgren A, Hörstedt P, Hammarström S, Hernell O (2004). Presence of bacteria and innate immunity of intestinal epithelium in childhood celiac disease.. Am J Gastroenterol.

[41] Report of Working Group of European Society of Paediatric Gastroenterology and Nutrition (1990). Revised criteria for diagnosis of coeliac disease.. Arch disease child.

[42] Marsh MN, Crowe PT (1995). Morphology of the mucosal lesion in gluten sensitivity.. Baillieres Clin Gastroenterol.

