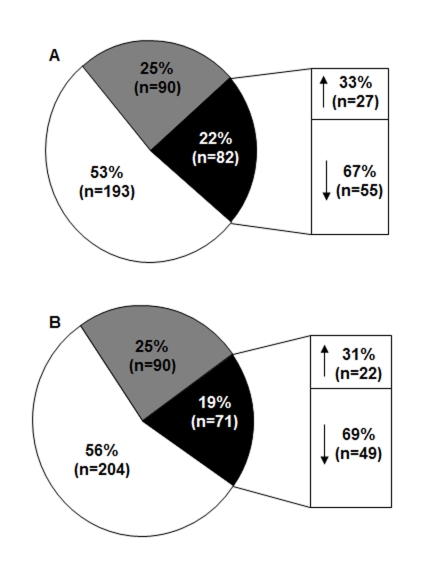# Correction: MicroRNA-449a Overexpression, Reduced NOTCH1 Signals and Scarce Goblet Cells Characterize the Small Intestine of Celiac Patients

**DOI:** 10.1371/annotation/7db01dfd-e044-41df-b1a8-9256ae11e4e2

**Published:** 2012-01-20

**Authors:** Marina Capuano, Laura Iaffaldano, Nadia Tinto, Donatella Montanaro, Valentina Capobianco, Valentina Izzo, Francesca Tucci, Giancarlo Troncone, Luigi Greco, Lucia Sacchetti

There is an error in Figure 1. The correct Figure 1 can be viewed here: 

**Figure:**