# Supplementary material for: MicroRNA-449a Overexpression, Reduced NOTCH1 Signals and Scarce Goblet Cells Characterize the Small Intestine of Celiac Patients
Source: PLoS One. 2011 Dec 15;6(12):e29094. doi: 10.1371/journal.pone.0029094 (PMC3240641; doi:10.1371/journal.pone.0029094)
Supplement: Materials and Methods S1 — (DOC) [file pone.0029094.s001.doc]

**Materials and Methods S1**

**miRNAs evaluation by TaqMan low density arrays**

Each TLDA Human MicroRNA Panel v1.0 card contained 365 preloaded human miRNA targets and two endogenous controls (small nucleolar RNAs: RNU48 (SNORD48) and RNU44 (SNORD44)). TLDAs were prepared in two-steps. In the first step, 800 ng of total RNA were reverse transcribed in eight multiplex reverse transcriptase (RT) reaction pools using stem loop RT primers specific for mature miRNA species. Then, each of the resulting eight cDNA pools was diluted, mixed with TaqMan Universal PCR master mix, and loaded into one of the eight fill ports on the TLDA microfluidic card. The card was centrifuged for 2 min at 1200 rpm to distribute samples to the multiple wells of the fill ports and sealed to prevent well-to-well contamination. Finally, the cards were processed on an ABI Prism 7900 HT apparatus (Applied Biosystems) and analyzed with the Sequence Detection System 2.3 software according to the delta-delta-Ct algorithm. The expression of miRNAs was first determined from the threshold cycle (Ct) values normalized to the expression of the reference RNU48. Then, the relative quantification (RQ) of each miRNA was calculated with the 2^-ΔΔCT^ algorithm and represented the miRNA fold change expression level measured in CD patients vs the mean level obtained in the controls.

**Immunohistochemistry**

Five μm thick sections were cut from the formalin-fixed tissue blocks, dewaxed in xylene analogs (Bio-Clear Bio-Optica, Milan, Italy) and re-hydrated with graded ethanol concentrations. The sections were incubated for 45 min at 97 °C in retrieval solution pH 9 (DAKO, S2367) (NOTCH1) or in citrate buffer pH 6 (DAKO, S2369) (HES1, KLF4, Ki67, MUC-2 and β-catenin) in order to retrieve immunogenicity. Endogenous peroxidase activity was blocked by immersing slides in 3% hydrogen peroxide methanol for 10 min. Aspecific antigen sites were blocked by incubating at room temperature for 30 min with background reducing components (DAKO). The primary antibodies used in the immunohistochemical staining were anti cleaved NOTCH1 rabbit monoclonal antibody (1:50 Cell Signaling Technology), anti-HES1 rabbit polyclonal antibody (1:1000 Abcam), anti-KLF4 monoclonal antibody (1:600 Santa Cruz), anti human Ki67 mouse monoclonal antibody (1:50 DAKO), anti mucin-2 (MUC-2) glycoprotein mouse monoclonal antibody (1:100 Leica Microsystems) and anti-β-catenin mouse monoclonal antibody (1:400 BD Transduction Laboratories). Tissue sections were incubated at room temperature for 1 h with primary antibodies. Staining was carried out with LSAB+System-HRP (DAKO); the signal was developed using diaminobenzidine (DAB) chromogen as substrate (DAKO). The tissue sections were then lightly counterstained with Mayer’s hematoxylin and cover-slipped.

**Oligonucleotides and plasmids**

Chemically modified double-stranded RNA molecules designed to pre-miR-449a microRNA precursor molecules and pre-miR-negative control were purchased from Ambion (Austin, TX, USA). The final concentrations of Pre-miR molecules and pre-miR negative control were 100nM and 300nM.

The plasmids used were pGL3-control (Promega Corp., Madison, WI, USA) encoding firefly luciferase and pRL-CMV encoding Renilla luciferase (Promega Corp., Madison, WI, USA). The 3’UTRs of NOTCH1 (179-185 bp 3’UTR) and of KLF4 (25-32 bp 3’UTR) genes, containing the miR-449a binding sites were obtained from genomic DNA by PCR with a sense and antisense primers carrying a XbaI restriction site, and cloned downstream to *Renilla* luciferase gene in pRL-CMV vector in XbaI site.

The above experiment was also performed after mutating the 3’UTR of KLF4 (three bases of seed region were changed by complementary bases) to further confirm the direct interaction among the 3’UTR of KLF4 and miR-449a, being the interaction miR-449a/3’UTR NOTCH1 recently validated by Marcet B et al [32].

**Cell culture, transfection, and luciferase assay**

The cell lines used were human HEK-293 maintained in α-MEM (minimum essential medium) supplemented with 10% fetal bovine serum and glutamine.

HEK-293 cells were seeded in 24-well plates with 500 µl of antibiotic-free medium the day before transfection to allow adherence and to reach 70–90% confluence at the time of transfection. The standard co-transfection mix was prepared for triplicate samples by adding 10 ng pGL3-control, 500 ng pRL-3’UTR or mutated 3’UTR and pre-miR in 150 µl of Opti-MEM I (Invitrogen); 3 µl lipofectamine 2000 (Invitrogen) was added separately in 150 µl Opti-MEM I. The two solutions were mixed and incubated at room temperature for 20-30 min, after which, 100 µl of the mix were added to each well. The final volume of medium plus transfection mix was 600 µl. Cells were incubated with the transfection mix for 6 h and the medium was then replaced with new fully supplemented culturing medium.
