# Supplementary figures and images for: MicroRNA-449a Overexpression, Reduced NOTCH1 Signals and Scarce Goblet Cells Characterize the Small Intestine of Celiac Patients
Source: PLoS One. 2011 Dec 15;6(12):e29094. doi: 10.1371/journal.pone.0029094 (PMC3240641; doi:10.1371/journal.pone.0029094)

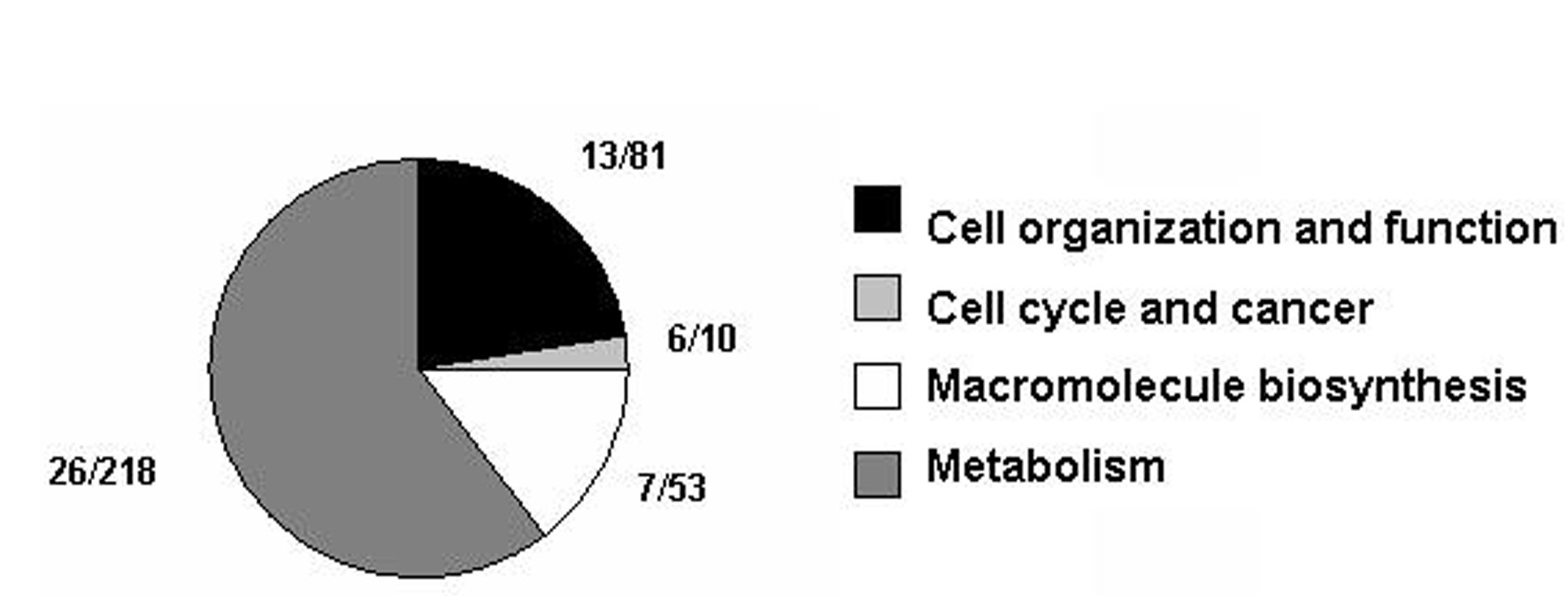

Supplement: Figure S1 — Bioinformatics analysis of miR-449a putative target genes. miR-449a putative target genes with most favorable context score, selected by bioinformatics, were sorted into pathways using GOTM (http://bioinfo.vanderbilt.edu/webgestalt/) and then combined into functional groups. (http://mirecords.biolead.org/interactions.php?species=Homosapiens&mirna_acc=hsa-miR-449a&targetgene_type=refseq_acc&targetgene_info=&v=yes&search_int=Search) (http://www.targetscan.org/cgibin/targetscan/vert_50/targetscan.cgi?species=Human&gid=&mir_sc=&mir_c=&mir_nc=&mirg=hsa-miR-449a). In each functional group are reported the genes belonging to NOTCH pathway/total gene number. (TIF) [file pone.0029094.s002.tif]

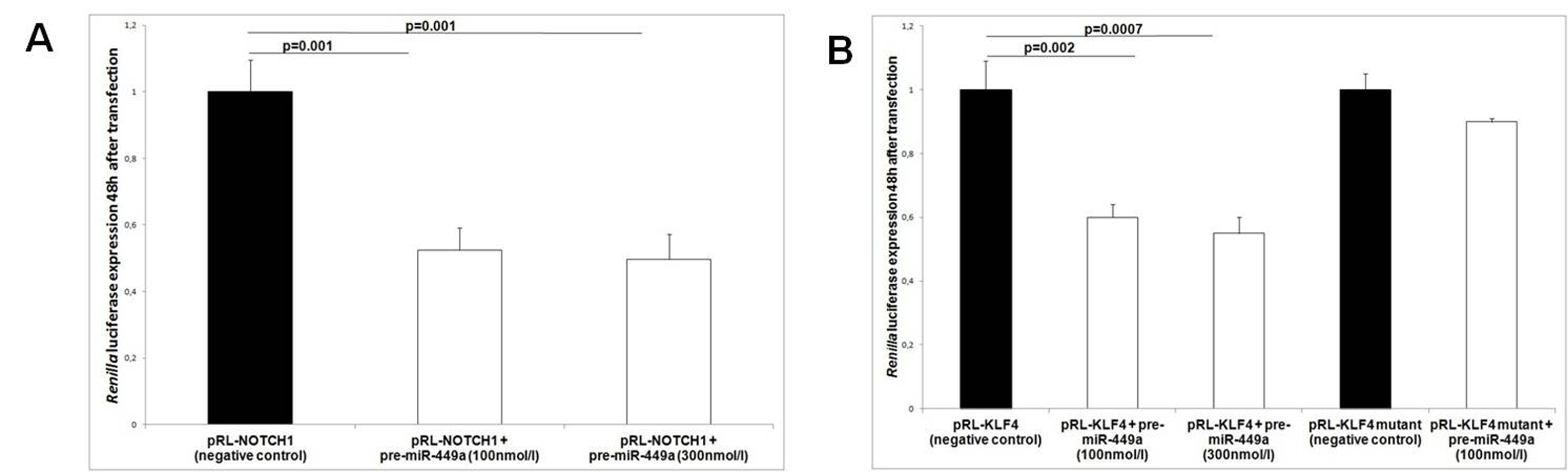

Supplement: Figure S2 — The luciferase assay confirms that miR-449a inhibits the expression of NOTCH1 and KLF4. In HEK293 cells co-transfected or with pRL-NOTCH1 vector (panel A) or with pRL-KLF4 vector (panel B), a pre-miR-449a concentration of 100 nmol/L was sufficient to significantly reduce (respectively, p = 0.001 and p = 0.002) Renilla luciferase activity versus control values. No inhibition of the Renilla luciferase expression was observed in mutant 3′UTR of KLF4-mRNA with miR-449a, so confirming the miR-449a/3′UTR KLF4-mRNA direct interaction (panel B). We didn't verify the interaction miR-449a/3′UTR NOTCH1 being this latter recently validated by Marcet B et al [32]. (TIF) [file pone.0029094.s003.tif]

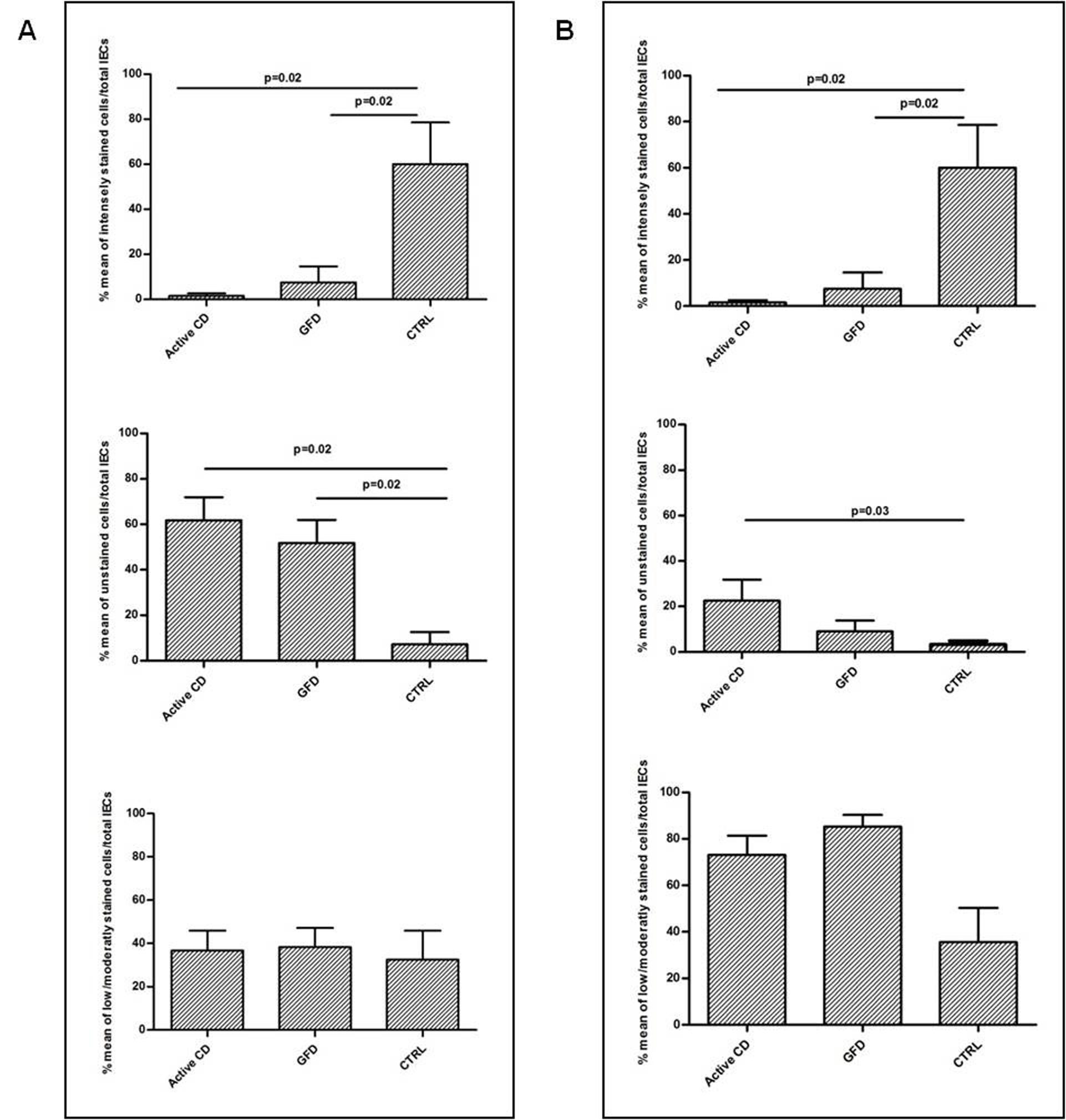

Supplement: Figure S3 — Automated Counts of NOTCH1 and HES1 stained/unstained cells. A. Automated counts of NOTCH1 stained/unstained cells (reported in Figure 2) in small intestine from CD patients (6 active CD and 6 GFD patients) and from controls (n = 4). Data are expressed as mean percent of intensely stained, low-moderately stained and unstained cells of the total intraepithelial cells (IECs) counted in ten crypts. The numbers of intensely stained and unstained cells were significantly (p = 0.02) higher and lower, respectively, in CTRL than in active CD and in GFD patients. B. Automated counts of HES1 stained/unstained cells (reported in Figure 3) in small intestine from CD patients (6 active CD and 6 GFD patients) and from controls (n = 4). Data are expressed as mean percent of intensely stained, low-moderately stained and unstained cells of the total intraepithelial cells (IECs) counted in ten crypts. The number of intensely stained cells was significantly higher in controls versus CD and GFD patients (p = 0.02) and the number of unstained cells was significantly lower in CTRL than in active CD patients (p = 0.03). (CTRL: controls; GFD: gluten free diet; CD: celiac disease). (TIF) [file pone.0029094.s004.tif]

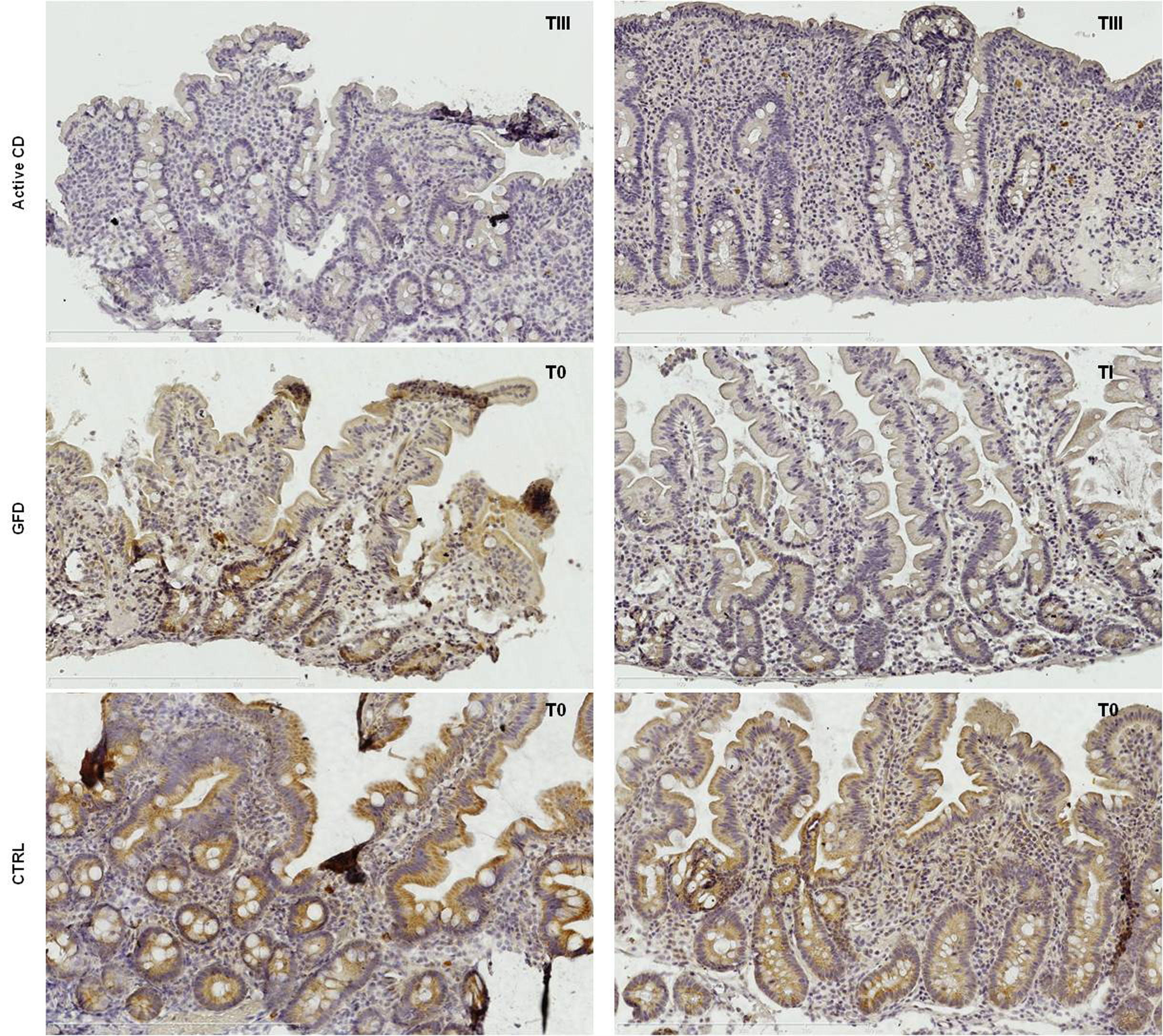

Supplement: Figure S4 — Other examples of NOTCH1 immunohistochemistry in CD patients. Examples of NOTCH1 immunohistochemistry in 4 CD patients (2 active CD: TIII Marsh stage and 2 GFD: TI and T0 Marsh stage) and 2 controls (T0 Marsh stage). The images show that the low expression levels of NOTCH1 in intestinal mucosa from CD patients were always present from TIII to T0 Marsh stage. (CTRL: controls; GFD: gluten free diet; CD: celiac disease). (TIF) [file pone.0029094.s005.tif]

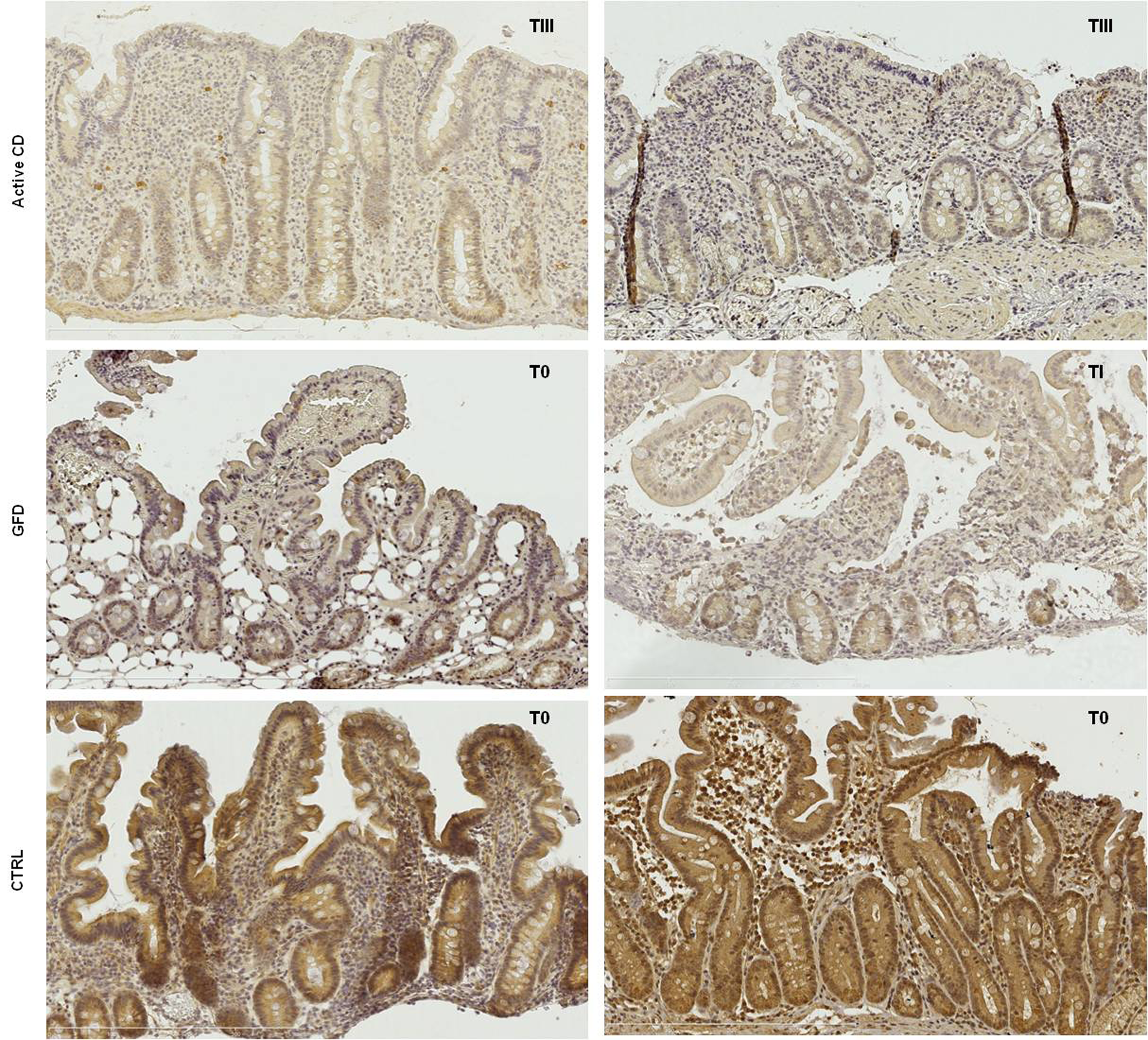

Supplement: Figure S5 — Other examples of HES1 immunohistochemistry in CD patients. Examples of HES1 immunohistochemistry in 4 CD patients (2 active CD: TIII Marsh stage and 2 GFD: TI and T0 Marsh stage) and 2 controls (T0 Marsh stage). The images show that the low expression levels of HES1 in intestinal mucosa from CD patients were always present from TIII to T0 Marsh stage. (CTRL: controls; GFD: gluten free diet; CD: celiac disease). (TIF) [file pone.0029094.s006.tif]

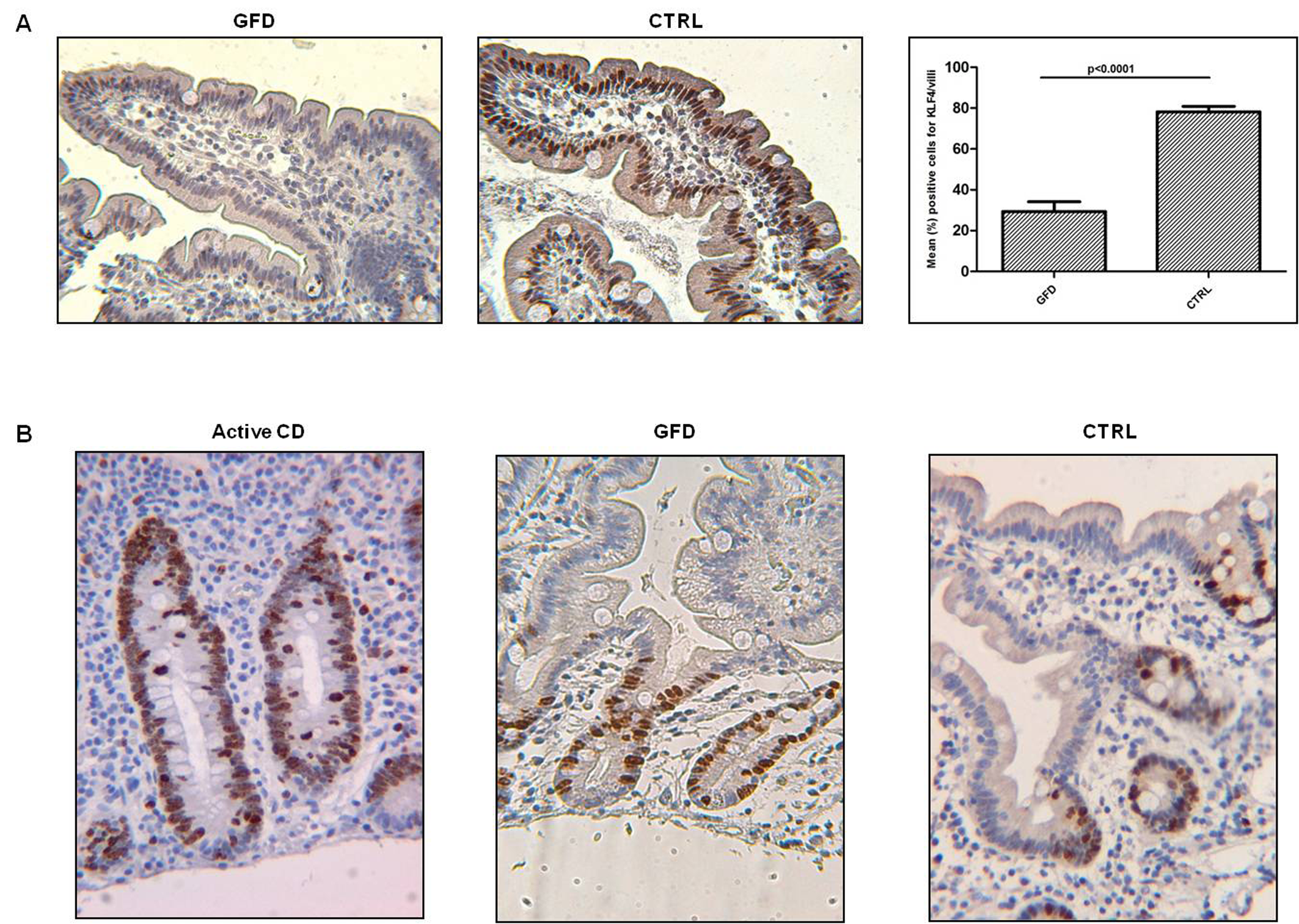

Supplement: Figure S6 — Decreased KLF4 and increased Ki67 expression in small intestine from CD patients compared with controls. A. KLF4 staining of small intestinal villi in GFD patients and Controls (Original magnification 20×). A statistically significant reduced KLF4-positive cells/villi were counted in GFD patients than in controls, respectively 29.0±5.0 vs 79.0±3.0 (mean±SEM) (p<0.0001). B. Increased Ki67 signal is present in small intestinal crypts of active CD, GFD patients than in controls (Original magnification 20×). (CTRL: controls; GFD: gluten free diet; CD: celiac disease). (TIF) [file pone.0029094.s007.tif]

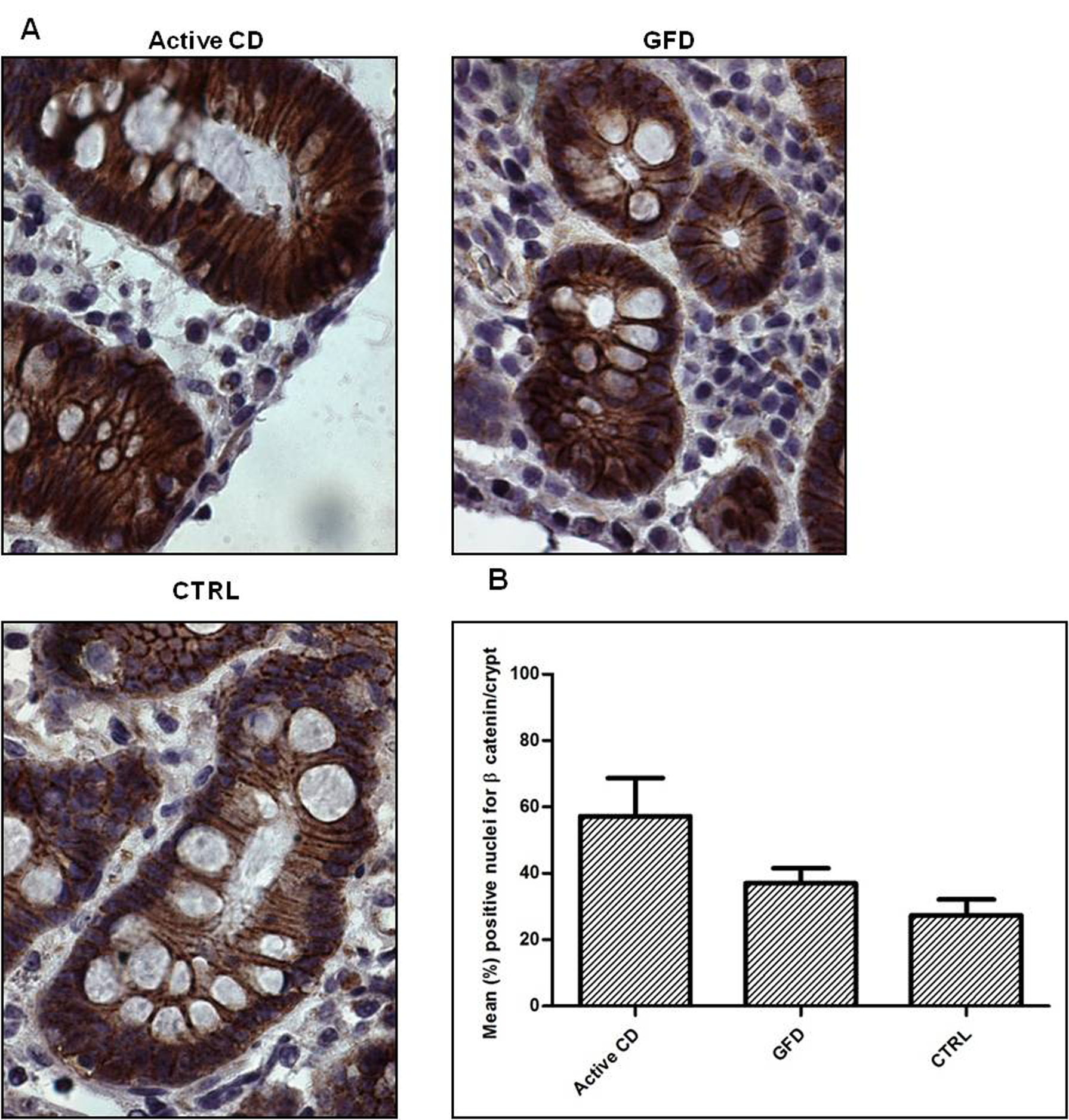

Supplement: Figure S7 — Increased expression of beta-catenin in small intestine from CD patients compared with controls. Immunostaining with beta-catenin in small intestinal crypts from active CD, GFD and controls. We counted the beta-catenin labeled nuclei. Similar counts of beta-catenin labelled nuclei were detected in the crypts of the small intestine in all groups. However, higher even if not statistical significant mean percentage counts (beta-catenin positive nuclei/crypt) were obtained in active CD and GFD than in controls, respectively 57.0±11.5 and 37.0±4.6 vs 27.0±4.6 (Original magnification 63×). (CTRL: controls; GFD: gluten free diet; CD: celiac disease). (TIF) [file pone.0029094.s008.tif]

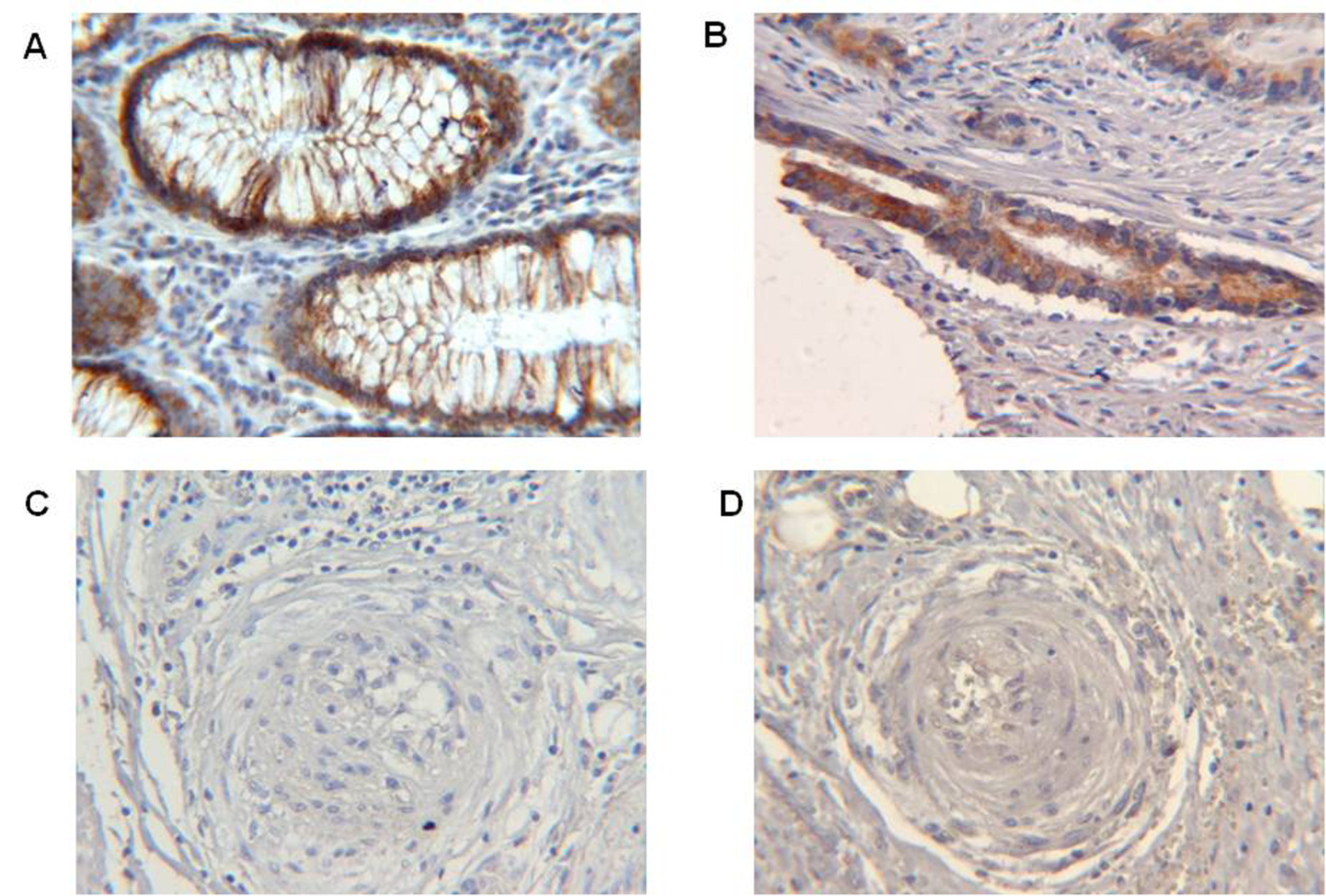

Supplement: Figure S8 — Specificity of NOTCH1 and HES1 signals by immunohistochemistry. Specificity controls of NOTCH1 and HES1 antibodies. Positive NOTCH1 (A) and HES1 (B) immunostaining signals obtained in human colon cancer and negative NOTCH1 (C) and HES1 (D) immunostaining signals obtained in human endothelial wall. (TIF) [file pone.0029094.s009.tif]
