# Supplementary material for: MicroRNA-449a Overexpression, Reduced NOTCH1 Signals and Scarce Goblet Cells Characterize the Small Intestine of Celiac Patients
Source: PLoS One. 2011 Dec 15;6(12):e29094. doi: 10.1371/journal.pone.0029094 (PMC3240641; doi:10.1371/journal.pone.0029094)
Supplement: Table S1 — MiRNAs differently expressed in active and GFD CD patients. (DOC) [file pone.0029094.s010.doc]

| Up regulated miRNAs | | Down regulated miRNAs | |
| --- | --- | --- | --- |
| Active CD patients | GFD CD patients | Active CD patients | GFD CD patients |
| **miR-182** | **miR-182** | **miR-105** | **miR-105** |
| **miR-196a** | **miR-196a** | **miR-124a** | **miR-124a** |
| **miR-330** | **miR-330** | **miR-135a** | **miR-135a** |
| **miR-449a** | **miR-449a** | **miR-189** | **miR-189** |
| **miR-492** | **miR-492** | **miR-202** | **miR-202** |
| **miR-500** | **miR-500** | **miR-219** | **miR-219** |
| **miR-503** | **miR-503** | **miR-299-5p** | **miR-299-5p** |
| **miR-504** | **miR-504** | **miR-323** | **miR-323** |
| **miR-644** | **miR-644** | **miR-379** | **miR-379** |
| miR-18a | miR-184 | **miR-380-5p** | **miR-380-5p** |
| miR-187 | miR-190 | **miR-409-5p** | **miR-409-5p** |
| miR-196b | miR-193b | **miR-412** | **miR-412** |
| miR-213 | miR-205 | **miR-512-3p** | **miR-512-3p** |
| miR-223 | miR-338 | **miR-566** | **miR-566** |
| miR-337 | miR-422a | **miR-576** | **miR-576** |
| miR-383 | miR-422b | **miR-600** | **miR-600** |
| miR-424 | miR-489 | **miR-614** | **miR-614** |
| miR-425 | miR-490 | **miR-616** | **miR-616** |
| miR-432 | miR-518d | **miR-618** | **miR-618** |
| miR-554 | miR-524 | **miR-631** | **miR-631** |
| miR-565 | miR-591 | **miR-659** | **miR-659** |
| miR-575 | miR-627 | miR-30a-3p | miR-27b |
| miR-589 |  | miR-30b | miR-17-3p |
| miR-597 |  | miR-30c | miR-99a |
| miR-630 |  | miR-30e-3p | miR-99b |
| miR-639 |  | miR-31 | miR-100 |
| miR-656 |  | miR-34b | miR-105 |
|  |  | miR-96 | miR-125a |
|  |  | miR-133a | miR-125b |
|  |  | miR-135b | miR-130a |
|  |  | miR-139 | miR-132 |
|  |  | miR-145 | miR-133b |
|  |  | miR-185 | miR-143 |
|  |  | miR-192 | miR-148a |
|  |  | miR-194 | miR-153 |
|  |  | miR-198 | miR-193a |
|  |  | miR-199a | miR-203 |
|  |  | miR-204 | miR-376a |
|  |  | miR-217 | miR-383 |
|  |  | miR-224 | miR-410 |
|  |  | miR-369-3p | miR-411 |
|  |  | miR-422b | miR-432 |
|  |  | miR-485-3p | miR-433 |
|  |  | miR-509 | miR-518b |
|  |  | miR-515-3p | miR-589 |
|  |  | miR-520h | miR-630 |
|  |  | miR-542-5p | miR-622 |
|  |  | miR-548d | miR-639 |
|  |  | miR-556 | miR-650 |
|  |  | miR-579 |  |
|  |  | miR-606 |  |
|  |  | miR-608 |  |
|  |  | miR-624 |  |
|  |  | miR-651 |  |
|  |  | miR-653 |  |
|  |  |  |  |
|  |  |  |  |
|  |  |  |  |
|  |  |  |  |
|  |  |  |  |
|  |  |  |  |
|  |  |  |  |

**Table S1.**  MiRNAs differently expressed in active and GFD CD patients.
